# Genome Sequences of Bacteriophages KaiHaiDragon and OneinaGillian, Isolated from Microbacterium foliorum in Riverside, California

**DOI:** 10.1128/MRA.00002-19

**Published:** 2019-04-11

**Authors:** Gillian Miller, Steven Tran, Rhiannon Abrahams, Daniel Bazan, Ethan Blaylock, Jessica Choi, Shannon Grewal, Elmira Hernandez, Daniel Kim, Kay Kim, Yumin Lee, Meagan Lopez, Rachel Specht, Nancy Kalaj, Arun Muthiah, Natasha Dean, Arturo Diaz

**Affiliations:** a Biology Department, La Sierra University, Riverside, California, USA; Broad Institute of MIT and Harvard University

## Abstract

KaiHaiDragon and OneinaGillian are two bacteriophages which have been recovered from soil samples using the bacterial host Microbacterium foliorum. Their genome lengths are 52,992 bp and 61,703 bp, with 91 and 104 predicted open reading frames, respectively. KaiHaiDragon belongs to cluster EC, and OneinaGillian is a member of cluster EG.

## Abstract (precis)

KaiHaiDragon and OneinaGillian are two bacteriophages which have been recovered from soil samples using the bacterial host Microbacterium foliorum. Their genome lengths are 52,992 bp and 61,703 bp, with 91 and 104 predicted open reading frames, respectively.

## ANNOUNCEMENT

To increase knowledge regarding bacteriophage diversity, La Sierra University’s chapter of the Science Education Alliance-Phage Hunters Advancing Genomics and Evolutionary Science (SEA-PHAGES) program (1) collected soil samples from Riverside, California, and isolated bacteriophages by directly plating the environmental samples on a bacterial lawn of Microbacterium foliorum NRRL B-24224. The genomes of two of the isolated phages, KaiHaiDragon and OneinaGillian, were selected by the class for sequencing and annotation.

DNA was isolated from high-titer phage lysates (>5 × 10^9^ PFU/ml) generated after two rounds of purification using the Wizard DNA cleanup system (Promega, catalog number A7280), and libraries were prepared using an NEB Ultra II FS kit at the Pittsburgh Bacteriophage Institute. The libraries were run on an Illumina MiSeq instrument, yielding at least 700,000 250-base paired-end reads per sample. Raw reads were assembled by using Newbler (version 2.6) and Consed (version 29) (2) into a single contig for each phage, with 1,039-fold and 2,049-fold coverage for KaiHaiDragon and OneinaGillian, respectively. The genome sequences were manually annotated with the assistance of DNA Master (http://cobamide2.bio.pitt.edu/computer.htm), ARAGORN (3), BLASTP (4), HHpred (5), and Phamerator (6), as outlined in the SEA-PHAGES bioinformatics guide (https://seaphagesbioinformatics.helpdocsonline.com/home).

Phages were assigned to clusters based on genomic sequence similarity, genomic synteny, and phylogenetic analysis using the Actinobacteriophage Database (PhagesDB) and the Phamerator software with default settings (6, 7). KaiHaiDragon was assigned to cluster EC, and OneinaGillian was assigned to cluster EG. KaiHaiDragon’s genome is circularly permutated, with a length of 52,992 base pairs. The genome has a 68.9% G+C content and encodes 91 genes. Interestingly, all of KaiHaiDragon’s open reading frames (ORFs) are transcribed in the forward direction. Functions were assigned to 30 of the predicted genes, and no tRNA genes were identified. Of note, KaiHaiDragon gp83 was found to encode an RNA polymerase sigma factor.

The OneinaGillian genome has a G+C content of 67.1% and is 61,703 base pairs long, and each terminus has 203-base pair direct terminal repeats. Average nucleotide identity (ANI) was determined by OrthoANI (8). OneinaGillian is only 72.1% and 70.49% identical, respectively, to Squash and Hyperion, which are also phages assigned to cluster EG. The beginning and the end of the genome of OneinaGillian are highly dissimilar to those of Squash and Hyperion due to the presence of 55 orphans, genes without detectable homologs in other sequenced phages, located in these regions. In comparison, Squash and Hyperion are 90.75% identical to each other. Out of a total of 104 genes in the OneinaGillian genome, only 29 genes were assigned a function, including a tRNA. OneinaGillian is organized with its virion structure and assembly genes in the leftmost 31 kbp of the genome, with a centrally located lysin A (gp45) and membrane protein genes. The nonstructural genes are present to the right of the lysin A gene, while the rightmost portion of the genome contains mostly orphan genes. Additional details on OneinaGillian and KaiHaiDragon can be found in the Actinobacteriophage Database (7).

### Data availability.

The GenBank and SRA accession numbers are MH590600 and SRX5352621 for KaiHaiDragon and MH727556 and SRX5352620 for OneinaGillian, respectively.
